# Retrospective Evaluation of Fulvestrant Efficacy and Clinical Results in Patients Using Fulvestrant

**DOI:** 10.7759/cureus.35748

**Published:** 2023-03-04

**Authors:** Engin Kut, Serkan Menekse

**Affiliations:** 1 Medical Oncology, Manisa State Hospital, Manisa, TUR

**Keywords:** hormone receptor-positive, hormone receptor-positive breast cancer, estrogen receptor (er), breast cancer, real-life data, fulvestrant

## Abstract

Aim: Fulvestrant is a drug used in the treatment of metastatic hormone receptor-positive breast cancer (mHRPBC). Although clinical trials have shown the efficacy of fulvestrant, real-life data are limited and data from clinical trials and real-life settings sometimes may be seen differently. Therefore, we retrospectively reviewed mHRPBC patients followed in our center and taking fulvestrant to evaluate the efficacy and clinical outcomes of the drug and also to identify factors affecting the efficacy and clinical outcomes of fulvestrant.

Materials and methods: Patients who were followed up with the diagnosis of metastatic breast cancer between 2010 and 2022 and using fulvestrant were retrospectively analyzed.

Results: The median progression-free survival (PFS) time was 9 [95% confidence interval (CI): 7.13-10.18] months and the median overall survival time was 28 (95% CI: 22.53-34.93) months. According to multivariate analyses, PFS was associated with age (p=0.041), body mass index (BMI) (p=0.043), brain metastasis (p=0.033), fulvestrant line (p=0.002), and use of pre-fulvestrant chemotherapy (p=0.032).

Conclusion: Fulvestrant is an effective drug in mHRPBC. Fulvestrant is more effective in patients whose BMI index is under 30, without brain metastases, without prior chemotherapy, under 65 years of age, and used fulvestrant in the early treatment line. The efficacy of fulvestrant may vary according to age and BMI.

## Introduction

Breast cancer is cancer with the highest incidence and mortality rate among women across the world [1]. The incidence and mortality rates of breast cancer have increased in the last three decades, and it is predicted that this increasing trend will continue in parallel with the growing adoption of Western lifestyles, especially in developing countries [2]. Metastatic hormone receptor-positive breast cancer (mHRPBC) and human epidermal growth factor receptor 2 (HER2)-negative diseases constitute 60%-70% of all breast cancers [2-3]. Fulvestrant is a selective estrogen receptor down regulator that binds to estrogen receptors with approximately 100 times greater than tamoxifen [3]. In the literature, the CONFIRM study reported that the efficacy of 500 mg fulvestrant was higher than 250 mg, and the subsequent phase II FIRST and phase III FALCON studies revealed that fulvestrant was more effective than first-line aromatase inhibitors in mHRPBC in postmenopausal women [4-6]. Following these studies, the National Comprehensive Cancer Network guideline recommended fulvestrant as a monotherapy option in patients with mHRPBC [7]. However, data reported by clinical trials may differ from the cases encountered in routine clinical settings. For example, there may be racial differences in drug metabolism on a regional basis. Although the number of visceral and non-visceral metastasis seen was similar, various factors, such as patients’ performance score, tumor burden, number of metastasis, concomitant comorbid diseases, and regional-racial polymorphism in drug metabolism may be different. This may sometimes result in discrepancies between data obtained from clinical studies and real-life data. Actual experience with treatment agents and regimens may assist physicians in their daily clinical practice.

There are few real-life data on fulvestrant in the literature. Therefore, we retrospectively reviewed mHRPBC patients who were followed up in our center and used fulvestrant and evaluated the efficacy and clinical outcomes of patients who used fulvestrant, and determined the factors affecting the efficacy and clinical outcomes of fulvestrant. 

## Materials and methods

Study populations

Post-menopausal mHRPBC patients who were followed up at the medical oncology clinic, using fulvestrant were retrospectively screened. The study included patients aged 18 years and older, who developed metastasis while taking tamoxifen and/or aromatase inhibitors in the adjuvant period (adjuvant hormonal therapy used for at least 12 months) or were detected to have metastasis at the time of diagnosis. All the patients had histologically confirmed mHRPBC and used 500 mg fulvestrant (on days 0, 14, and 28, followed by every 28 days) until treatment was discontinued due to progression, toxicity, or mortality.

Overall survival (OS) times of the patients are defined as the time from the date of diagnosis to mortality or the last follow-up for the surviving patients. Progression-free survival (PFS) was determined as the time from the beginning of fulvestrant use to disease progression or mortality from any cause. PFS, OS, clinical response, adverse events, and clinical response to subsequent post-fulvestrant therapy were investigated. Body mass index (BMI) was calculated by dividing the body weight by the square of height (kg/m²). The patients were classified into two groups according to their BMI values (<30 and ≥30). The primary endpoint was PFS, and the secondary endpoint was factors affecting PFS. The Response Evaluation Criteria in Solid Tumors (RECIST) were used to evaluate clinical response. The best overall response was determined according to the following criteria: complete response (CR), stable disease (SD), partial response (PR), and progressive disease (PD). The clinical benefit rate (CBR) of the patients was calculated by summing the three response rates (CR + PR + SD), and the overall response rate (ORR) was calculated by summing the partial and complete responses.

Statistical analysis 

Descriptive statistics were presented as numbers and percentages for categorical variables, and median with minimum and maximum values and mean ± standard deviation for numerical variables. Survival analysis was performed with the Kaplan-Meier method. Significant variables in the univariate analysis were introduced into a multivariate Cox model. The p-value <0.05 was considered significant in all statistical analyses.

## Results

A total of 132 patients were evaluated. The median age of the patients was 60 (48-81) years (Table 1). The median follow-up time was 38 (6-133) months. Thirty-five (26.5%) patients were metastatic at the time of diagnosis and 97 (73.5%) developed metastasis during follow-up while under treatment. Estrogen receptor was positive in all patients, and progesterone receptor was positive in 116 (87.9%) patients. All the patients included in the study were HER2-negative. The median estrogen receptor percentage was 82% (12%-100%) and the median progesterone receptor percentage was 70% (0-100%). All the patients had metastasis in more than one site during fulvestrant use. Ninety-nine (75%) patients received chemotherapy and endocrine therapy in the metastatic stage, while 33 (25%) patients received only endocrine therapy.

**Table 1:** Demographic and clinicopathological characteristics of the patients. BMI, body mass index;  LN, lymph node; CR, complete response; PR, partial response; SD, stable disease; PD, progressive disease; HER2, human epidermal growth factor receptor 2

Parameters		Number (n)	Percentage (%)
Age	<65 years	89	67.4
	≥65 years	13	13.6
BMI	<30	70	53
	≥30	62	47
Type of metastasis	Synchronous	35	26.5
	Metachronous	97	73.5
Histology	Invasive ductal	115	87.1
	Medullar	11	9.8
	Lobular	3	2.3
	Papillary	1	o.8
Grade	Grade 1	8	6.1
	Grade 2	87	65.9
	Grade 3	37	28
Lymphovascular invasion	Positive	81	61.4
Perineural invasion	Positive	74	56.1
Progesterone receptör	Positive	116	87.9
Estrogen receptor	Positive	132	100
HER2	Negative	132	100
Metastasis site	Bone	107	81.1
	Brain	20	15.2
	Lung	51	58.6
	LN	76	57.6
	Liver	48	36.4
Fulvestrant line	First line	29	22
	Second line	82	62.1
	Third or more	21	15.9
Treatment response	CR	3	2.3
	PR	19	14.4
	SD	50	37.9
	PD	60	45.4

**Figure 1:**
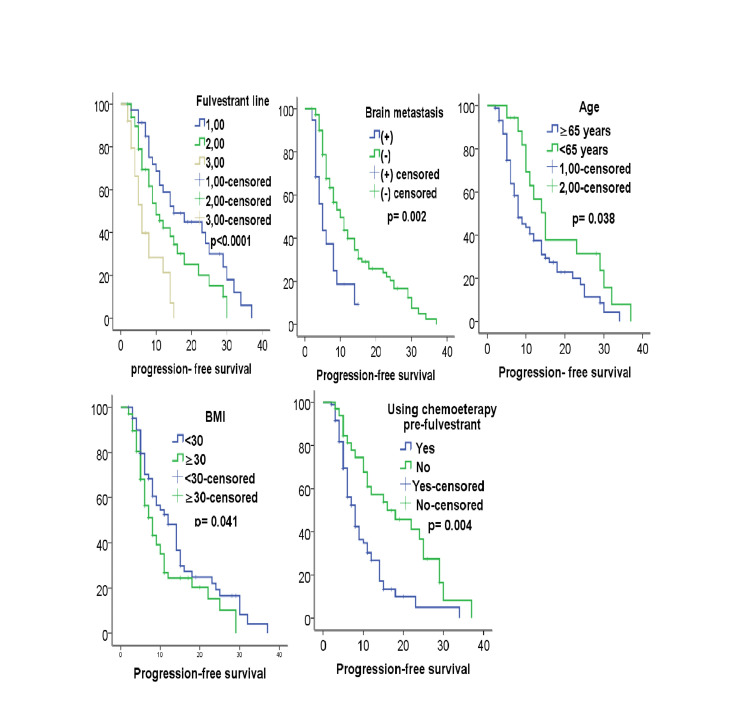
Kaplan-Meier curves show progression-free survival for fulvestrant line, brain metastasis, age, BMI, using chemotherapy pre-fulvestrant. BMI, body mass index

At the time of conducting statistical analyses for the study, 36 (27 alive patients were still alive, and 95 (72%) had died). Five (3.8%) patients continued to take fulvestrant as monotherapy. Fulvestrant was not discontinued in any of the patients due to drug toxicity. No side effects of grade 2 and above were observed. None of our patients had a BMI below 18 or above 40. Seventy (53%) patients had a BMI below 30 and 62 (47%) patients had a BMI equal to or above 30. The median PFS was 9 [95% confidence interval (CI): 7.13-10.18] months, and the median OS time was 28 (95% CI: 22.53-34.93) months. The median PFS times of the patients using fulvestrant as the first-, second-, and third or more line treatment were 12 (95% CI: 10.6-13.9), 9 (95% CI: 6.26-11.74), and 6 (95% CI: 3.66-8.34) months, respectively. PFS was associated with age (p=0.038), BMI (p=0.041), brain metastasis (p=0.002), lymphovascular invasion (p=0.042), perineural invasion (p=0.02), grade (p=0.02), fulvestrant line (p<0.0001), progesterone level (p=0.024), and use of chemotherapy before fulvestrant (p=0.004) in the univariate analysis, and age (p=0.041), BMI (p=0.043), brain metastasis (p=0.033), fulvestrant line (p=0.002), and use of chemotherapy before fulvestrant (p=0.32) according to the multivariate analysis (Table 2, Figure 1).

**Table 2:** Univariate and multivariate analyses of overall survival. HR, hazard ratio; CI, confidence interval; BMI, body mass index; LN, lymph node; LVI, lymphovascular invasion; PNI, perineural invasion

	Univariate analysis (HR, 95% CI)	p value	Multivariate analysis (HR, 95% CI)	p value
Age (<65 vs ≥65 years)	1.82 (1.41-3.29)	0.038	1.92(1.02-3.58)	0.041
BMI (<30 vs ≥30)	1.54 (1.21-2.35)	0.041	1.78( 1.07-2.97)	0.043
Tumor grade	1.54 (1.088-.1.872)	0.02	1.89 (1.01-3.56)	0.47
LVI	1.55 (1.017-2.540)	0.042	1.22 (0.72-2.279	0.40
PNI	1.63 (1.078-2.46)	0.020	1.38 (0.78-2.42)	0.27
Progesterone	2.39 ((1.32-4.26)	0.004	1.32 (0.52-3.29)	0.55
Estrogen	0.94 (0.98-1.00)	0.50		
Bone metastasis	-0.67 (0.39-1.12)	0.12		
Lung metastasis	-0.69 (0.46-1.06)	0.09		
Liver metastasis	1.41 (0.96-2.25)	0.77		
Brain metastasis	2.46 (1.39-4.33)	0.002	1.96 (1.55-3.49)	0.033
LN metastasis	1.22 (0.802-1.85)	0.355		
Histology (ductal vs other type)	-0.72 (0.100-5.217)	0.571		
Fulvestrant line		<0.001		0.002
First vs second line	1.73 (1.25-3.03)	0.028	1.72(1.07-2.65)	0.015
First line vs third or more	4.19 (2.18-8.03)	<0.001	2.65(1.24-5.85)	<0.001
Use of pre-fulvestrant chemotherapy	2.06 (1.27-3.37)	0.004	1.89 (1.05-3.39)	0.032

## Discussion

In this study, we investigated the efficacy and clinical outcomes of fulvestrant and the reasons affecting the efficacy and clinical outcomes of fulvestrant in mHRPBC patients using fulvestrant. We found that the efficacy of fulvestrant was similar to clinical trials and previous real-life data, but that this efficacy of fulvestrant may vary with age and BMI. We determined the median PFS as 9 months, ORR as 16.7%, and CBR as 50.25%. In our study, median PFS was longer than that reported in the CONFIRM study, similar to that of the FALCON study, and consistent with the findings reported by Palumbo et al. [8] and Lv et al. [9] in studies using real-life data.

In our study, fulvestrant was also effective in the first, second, and third. However, the PFS was longer on the first line than on the other lines. These findings support the use of fulvestrant as a first-line treatment, similar to the studies with clinical trials and real-life data by Palumbo et al., Lv et al., and Andrahennadi et al. [4-10]. Using multiline endocrine therapy before fulvestrant may change the hormone receptor status and cause the development of resistance mechanisms to endocrine therapy [9]. This may explain why patients using the advanced line have a shorter PFS than those using the first line. Additionally, fulvestrant is a more potent drug than tamoxifen and aromatase inhibitors. The use of a more potent agent in the first-line treatment may contribute to a deeper objective response and greater and longer-lasting clinical benefit. All of these may be a reason why patients using fulvestrant in the first line have longer PFS.

In our study, our patients received fulvestrant as monotherapy. Although fulvestrant monotherapy was more effective in early lines in our study, according to recent studies, CDK4/6 inhibitors and their combinations (combinations with fulvestrant and aromatase inhibitors) are recommended as the standard for the treatment of mHRPBC in patients who have never received prior treatment [11]. Treatment with CD4/6 inhibitors has a more favorable effect on both ORR and PFS in patients [11]. However, fulvestrant can be used as first-line monotherapy in the early stages in patients who cannot tolerate CDK4/6 or cannot use CDK4/6 inhibitors due to oligometastatic disease, advanced age, comorbid diseases, fragility, and cytopenia [10-11].

Breast cancer is the most common cancer among women, and its incidence increases with age. In general, 40% of all breast cancers occur over the age of 65 years. Older patients show some differences from younger patients. Advanced-age breast cases tend to be more mHRPBC than younger patients [2, 12]. In addition, more favorable breast cancer subtypes, such as papillary carcinoma are observed in elderly female patients [2, 12]. Compared to younger patients, elderly patients also have more comorbid diseases, physiological changes, decreased renal clearance, reduced body defense systems, fragility, drug interactions, and changes in drug metabolism. Therefore, treatment response and fulvestrant efficacy may be different in this patient population. In our study, fulvestrant resulted in longer PFS in patients younger than 65 years. In the study by Kawaguchi et al., the median age of the patients was 60 years [13]. Kawaguchi et al. reported that the efficacy of fulvestrant may vary in patients above and below 60 years of age [13]. To our knowledge, there is no other study in the literature reporting that the efficacy of fulvestrant may vary with age except the study of Kawaguchi et al. and our study [13]. Therefore, according to our study and the results reported by Kawaguchi et al., age should be considered when evaluating the efficacy of fulvestrant [13].

Studies conducted in recent years have found a relationship between obesity and many cancer prognoses [14-17]. This is attributed firstly to cardiovascular complications caused by obesity itself and secondly to chronic inflammation caused by obesity, immune system activation, the release of pro-inflammatory cytokines and neoangiogenesis due to these pro-inflammatory cytokines, and changes in the tumor microenvironment. There are no prospective studies examining the relationship between the efficacy of fulvestrant and body weight, body surface area, or BMI. Fulvestrant dose does not change according to weight. Fulvestrant 500 mg is administered to all patients at the same dose. In a retrospective study by Gevorgyan et al. evaluating a small number of patients, it was reported that increased BMI had a negative effect on treatment outcomes [18]. In our study, we found that patients with a BMI below 30 had longer PFS. However, there were no patients in our study with a BMI below 18 or above 40. To our knowledge, there is no other study in the literature reporting that the efficacy of fulvestrant may vary with BMI except the study of Gevorgyan et al. and our study [18]. Therefore, according to our study and the results reported by Gevorgyan et al., BMI should be considered when evaluating the efficacy of fulvestrant [18].

Although the use of combinations of CDK4/6 inhibitors (fulvestrant or letrozole) or fulvestrant as monotherapy in the first-line treatment of mHRPBC increases the treatment response of patients [11, 19]. The most important problem in the treatment of mHRPBC patients is endocrine therapy resistance [19]. There is no known biomarker that predicts who has endocrine therapy resistance or who will benefit from endocrine therapy [19]. Resistance mechanisms are being overcome with the development of drugs such as CDK4/6 inhibitors (palbociclib, ribociclib, abemaciclib), everolimus (acting through inhibition of the PI3K/Akt/mTOR pathway), and combinations of these drugs with other endocrine therapy options in daily practice [11, 19]. Since there is no biomarker that can help predict resistance to endocrine therapy and the efficacy of endocrine therapy, knowing that fulvestrant efficacy may vary according to age and BMI may help us to decide on the treatment of a specific group of mHRPBC.

In the literature, it has been reported that low-grade tumors respond better to endocrine treatments. In addition, previous studies have shown that tumor grade and the presence of lymphovascular and perineural invasion are associated with breast cancer prognosis [20-21]. In contrast, we did not observe a significant relationship between clinical response and tumor grade, lymphovascular invasion, and perineural invasion. This may be because although almost all of our patients had breast biopsies, the number of biopsies obtained from metastasis was fewer. The characteristics of tumors in metastasis and primary tumors may be different due to tumor heterogeneity, or an optimal evaluation may be difficult since the biopsy materials taken are much smaller compared to the surgical material samples.

Some studies have reported a significant relationship between the type of organ metastasis and survival. Palumbo et al. observed a relationship between liver metastasis and survival, and Arıcı et al. detected a relationship between liver and brain metastasis and survival, while Lv et al. found no relationship between visceral organ metastasis and survival [8-9, 22]. In the current study, we determined that the patients with brain metastasis had shorter PFS.

The reason for these discrepancies between the studies may be related to the differences in the number of patients, tumor burden, number of metastasis, performance scores, and other accompanying organ metastasis.

The limitations of our study include its single-center, small number of patients, and retrospective nature. However, our study is an important study in that it is one of the few studies to present real-life data on the efficacy of fulvestrant with PFS and response rates, and similar to clinical trials, it shows that early use of fulvestrant has a greater contribution to PFS, and also reports for the first time that the efficacy of fulvestrant may vary according to BMI and age.

## Conclusions

Fulvestrant is an effective drug in postmenopausal mHRPBC cases. The efficacy of fulvestrant may vary according to age and BMI. Fulvestrant is more effective in patients whose BMI is under 30, without brain metastases, if not receiving chemotherapy before, below 65 years of age, and use as an agent in the early lines.
